# Draft Genome Sequence of the Plant Growth-Promoting Bacterium Bacillus subtilis Strain BZR 517, Isolated from Winter Wheat, Now Reclassified as Bacillus velezensis Strain BZR 517

**DOI:** 10.1128/MRA.00853-20

**Published:** 2020-10-01

**Authors:** Vitaly V. Radchenko, Ilya Y. Vasilyev, Elena V. Ilnitskaya, Alexey V. Garkovenko, Anzhela M. Asaturova, Natalia S. Tomashevich, Alexander E. Kozitsyn, Alexander V. Milovanov, Tatiana V. Grigoreva, Margarita V. Shternshis

**Affiliations:** a M. M. Shemyakin and Y. A. Ovchinnikov Institute of Bioorganic Chemistry, Russian Academy of Sciences, Moscow, Russia; b Kuban State Agrarian University named after I. T. Trubilin, Krasnodar, Russia; c Kazan (Volga Region) Federal University, Kazan, Russia; d All-Russian Research Institute of Biological Plant Protection, Krasnodar, Russia; University of Arizona

## Abstract

Bacillus velezensis strain BZR 517 is a prospective plant growth-promoting rhizobacterium with known biocontrol properties, which may be used to improve soil quality. The genome sequencing was conducted as part of new biological agent development in order to determine the biocontrol potential of the strain, including the production of biologically active compounds.

## ANNOUNCEMENT

The species Bacillus subtilis, whose genome was first sequenced in 1997 by Kunst, was later classified as a complex taxon containing many phylogenetically close branches (1). Bacillus velezensis, which was historically known as Bacillus amyloliquefaciens, Bacillus methylotrophicus, and Bacillus oryzicola (2), was characterized as a producer of a great variety of biologically active compounds (3) with a wide spectrum of possible applications, including in plant biotechnology and agricultural industries. In particular, vital spores of B. amyloliquefaciens FZB42 are included in the commercially available biostimulating microbial inoculant RhizoVital 42 (Andermatt Biocontrol AG, Switzerland) (4, 5), and living B. velezensis cultures are the base of the commercial fungicide Botrybel (Agricaldes, Spain) (6).

B. velezensis strain BZR 517 was previously isolated from the roots of winter wheat grown in the Krylovsky district, Krasnodar Region, Russia, and was described as B. subtilis BZR 517 based on its morphological features and 16S rRNA gene sequence analysis. It was placed in the Departmental Collection of Useful Microorganisms for Agricultural Purposes (accession number RCAM 01728) as a plant growth stimulator (7), an effective producer of hydrolytic ferments, and a suppressor of common fungal phytopathogens, such as *Fusarium* spp., *Microdochium* spp., and *Pyrenophora* spp. (8, 9), with an absence of pathogenic properties for plants and homeothermic animals. It was also patented as a prospective biological agent for crop protection and control of phytopathogenic fungi (10, 11).

The B. velezensis BZR 517 whole-genome shotgun (WGS) sequencing was conducted as part of the research dedicated to its antimicrobial and fungicidal activities for the purpose of developing a relevant commercial biocontrol agent. Details of the biomaterial preparation were described earlier (12). The whole-genome DNA was isolated using the Nextera DNA Flex library preparation kit (Illumina, Inc., USA) and sequenced using the Illumina MiSeq platform; a total of 5,128,270 paired-end reads (median, 151 bp) were generated after quality filtering. The resulting WGS assembly contained 3,977,982 bp in 52 contigs, with expected coverage of 98.69× (compared to B. velezensis Hx05 [GenBank accession number CP029473.2], which was used as a reference), an *N*_50_ value of 341,123 bp, and a G+C content of 46.37%. Read preprocessing was performed using FastQC v.0.11.8 (https://github.com/s-andrews/FastQC), Trimmomatic v.0.39 (http://www.usadellab.org/cms/?page=trimmomatic), and Cutadapt v.2.5 (https://cutadapt.readthedocs.io/en/stable), and the genome was assembled with the SPAdes v.3.9.1 toolkit (https://cab.spbu.ru/software/spades). All software was deployed in separate Docker containers. The WGS assembly was annotated remotely on the NCBI Prokaryotic Genome Annotation Pipeline (PGAP) using the best-placed reference protein set and GeneMarkS-2+ annotation software rev. 4.10 (13). As reported, the BZR 517 WGS assembly sequence contained 3,884 coding sequences, 68 hypothetical proteins, 7 rRNAs, 97 tRNAs, and 5 noncoding RNAs.

### Data availability.

The complete project processing materials, including data, automation scripts, tools, relevant software launch parameters, and arguments, are available at the GitHub repository (https://github.com/ivasilyev/curated_projects/tree/master/vradchenko/lactobacillus_salivarius). The B. velezensis BZR 517 sequences obtained have been deposited in NCBI databases under the following accession numbers: PRJNA588983 (BioProject), SRX8162232 (SRA), and WKKV00000000 (GenBank).

## References

[1] Kunst F, Ogasawara N, Moszer I, Albertini AM, Alloni G, Azevedo V, Bertero MG, Bessières P, Bolotin A, Borchert S, Borriss R, Boursier L, Brans A, Braun M, Brignell SC, Bron S, Brouillet S, Bruschi CV, Caldwell B, Capuano V, Carter NM, Choi SK, Cordani JJ, Connerton IF, Cummings NJ, Daniel RA, Denziot F, Devine KM, Düsterhöft A, Ehrlich SD, Emmerson PT, Entian KD, Errington J, Fabret C, Ferrari E, Foulger D, Fritz C, Fujita M, Fujita Y, Fuma S, Galizzi A, Galleron N, Ghim SY, Glaser P, Goffeau A, Golightly EJ, Grandi G, Guiseppi G 1997 The complete genome sequence of the Gram-positive bacterium *Bacillus subtilis*. Nature 390:249–256. doi:10.1038/36786. 9384377

[2] Borriss R, Danchin A, Harwood CR, Médigue C, Rocha EPC, Sekowska A, Vallenet D 2018 *Bacillus subtilis*, the model Gram-positive bacterium: 20 years of annotation refinement. Microb Biotechnol 11:3–17. doi:10.1111/1751-7915.13043. 29280348 PMC5743806

[3] Chen XH, Koumoutsi A, Scholz R, Eisenreich A, Schneider K, Heinemeyer I, Morgenstern B, Voss B, Hess WR, Reva O, Junge H, Voigt B, Jungblut PR, Vater J, Süssmuth R, Liesegang H, Strittmatter A, Gottschalk G, Borriss R 2007 Comparative analysis of the complete genome sequence of the plant growth-promoting bacterium *Bacillus amyloliquefaciens* FZB42. Nat Biotechnol 25:1007–1014. doi:10.1038/nbt1325. 17704766

[4] Cawoy H, Bettiol W, Fickers P, Ongena M 2011 *Bacillus*‐based biological control of plant diseases, p 274–302. *In* Stoytcheva M (ed), Pesticides in the modern world: pesticides use and management. InTech, Rijeka, Croatia.

[5] Krebs B, Höding B, Kübart S, Workie MA, Junge H, Schmiedeknecht G, Grosch R, Bochow H, Hevesi M 1998 Use of *Bacillus subtilis* as biocontrol agent. I. Activities and characterization of *Bacillus subtilis* strains. J Plant Dis Prot 105:181–197.

[6] Romanazzi G, Feliziani E 2014 *Botrytis cinerea* (gray mold), p 131–146. *In* Bautista-Banos S (ed), Postharvest decay: control strategies. Elsevier, Amsterdam, Netherlands.

[7] Asaturova AM, Dubyaga VM 10 6 2015 Strain of bacteria *Bacillus subtilis* BZR 517 for obtaining biopreparation against phytopathogenic fungi. Russian patent RU2552146 C1.

[8] Sidorova TM, Asaturova AM, Homyak AI, Tomashevich NS 2019 Isolation and characterization of antifungal metabolites of *Bacillus subtilis* strains BZR 336g and BZR 517 using the modified bioauthography method. Agric Biol 54:178–185. doi:10.15389/agrobiology.2019.1.178eng.

[9] Sidorova TM, Asaturova AM, Homyak AI 2018 Biologically active metabolites of *Bacillus subtilis* and their role in the control of phytopathogenic microorganisms. Agric Biol 53:29–37. doi:10.15389/agrobiology.2018.1.29eng.

[10] Asaturova AM, Zhevnova NA, Pavlova MD, Dubyaga VM, Tomashevich NS, Khomyak AI, Tsygichko AA, Bondarchuk EY, Sidorova ТМ 2019 Efficiency of winter wheat seed inoculation by the Bacillus bacteria promising for the development of biopreparations. Grain Econ Russia 2:8–12. (In Russian.) doi:10.31367/2079-8725-2019-62-2-8-12.

[11] Asaturova AM, Homyak AI, Tomashevich NS, Pavlova MD, Zhevnova NA, Dubyaga VM, Kozitsin AY, Sidorova TM, Nadykta VD, Ismailov VY 2015 Conditions for the cultivation of new *Bacillus* bacteria being micro bioproduct producers. J Pure Appl Microbiol 9:2797–2804.

[12] Garkovenko AV, Vasilyev IY, Ilnitskaya EV, Radchenko VV, Asaturova AM, Kozitsyn AE, Tomashevich NS, Milovanov AV, Grigoreva TV, Shternshis MV 2020 Draft genome sequence of *Bacillus velezensis* BZR 336g, a plant growth-promoting antifungal biocontrol agent isolated from winter wheat. Microbiol Resour Announc 9:e00450-20. doi:10.1128/MRA.00450-20. 32703828 PMC7378027

[13] Tatusova T, DiCuccio M, Badretdin A, Chetvernin V, Nawrocki EP, Zaslavsky L, Lomsadze A, Pruitt KD, Borodovsky M, Ostell J 2016 NCBI Prokaryotic Genome Annotation Pipeline. Nucleic Acids Res 44:6614–6624. doi:10.1093/nar/gkw569. 27342282 PMC5001611

